# A qualitative study to explore the perspectives of key stakeholders regarding pharmaceutical pictograms in Pakistan

**DOI:** 10.1080/20523211.2025.2598481

**Published:** 2026-01-12

**Authors:** Kanza Arshad, Muhammad Atif, Wajiha Razzaq, Ali Arshad

**Affiliations:** a Department of Pharmacy Practice, Faculty of Pharmacy, The Islamia University of Bahawalpur, Bahawalpur, Pakistan; b Emergency Department, Tertiary Care Headquarter Hospital, Sadiqabad, Pakistan

**Keywords:** Pharmaceutical pictograms, patient communication, graphic images, pictorial symbols, health literacy, health communication tools, pictorial labelling

## Abstract

**Background:**

This qualitative study in Pakistan aims to gather perspectives from health regulators, pharmaceutical manufacturers, and healthcare professionals regarding the implementation of pharmaceutical pictograms.

**Methods:**

An exploratory qualitative study design was used to conduct this study across all provinces of Pakistan. The study adopted a constructivist approach to examine stakeholders’ subjective interpretations and to ensure methodological transparency, Consolidated Criteria for Reporting Qualitative Research (COREQ) guidelines were used as reporting guidelines. The data were collected from three main groups through in-depth, face-to-face interviews. The study participants were recruited using a purposive convenience sampling technique. Data analysis was conducted using an inductive thematic analysis approach to draw conclusions aligned with the study objectives.

**Results:**

A total of 57 respondents participated in the study, including 13 doctors, 15 pharmacists, 15 health regulators, and 14 manufacturers. Analysis of the data yielded five themes and sixteen subthemes. The five key themes were extracted including the current status of pharmaceutical pictograms, the impact of pharmaceutical pictograms, the significance of pharmaceutical pictograms on patients and the healthcare system, and challenges in the implementation of pharmaceuticals. The majority of the respondents demonstrated a better understanding of the term ‘pharmaceutical pictograms,’ but awareness of standardised systems (USP and FIP) was limited. However, several implementation challenges were identified, including a lack of government recognition and policy support, insufficient training and knowledge among healthcare professionals, and financial or resource constraints. Stakeholders emphasised national policy, training, and awareness to integrate pharmaceutical pictograms.

**Conclusion:**

The key stakeholders strongly favoured the implementation of pharmaceutical pictograms, recognising their potential to enhance patient comprehension, adherence, and overall safety in Pakistan. However, their adoption is limited by insufficient awareness, policy support, and resources. Addressing these challenges through national policies, professional training, and awareness initiatives is essential for the successful adoption of pictograms in the healthcare system.

## Background

Unsafe medication practices and medication errors are major contributors to adverse outcomes and preventable adverse events across the world, highlighting the critical importance of patient safety (Carroll et al., 2024). Patients’ ability to comprehend health-related information varies widely, making clear and accessible communication essential. Verbal explanations alone are often insufficient, particularly for patients with low health literacy or language barriers, highlighting the need for supplementary tools to enhance understanding (Ilardo & Speciale, 2020; Malhotra et al., 2023).

Pictograms are widely employed across diverse sectors, including healthcare, transportation, and marketing, as effective tools for conveying critical information. In the pharmaceutical context, the United States Pharmacopeia (USP) defines pharmaceutical pictograms as ‘standardized graphic images that help communicate drug treatment instructions, precautions, and/or warnings to patients and consumers’(Gutierrez et al., 2022). The use of pharmaceutical pictograms was first addressed by the United States (US) Pharmacopoeia Commission in 1987, followed by the introduction of a set of 29 pictograms in 1989 (Pedersen, 2019). In 1990, the International Pharmaceutical Federation (FIP) expanded this effort by creating a set of 75 pictograms, and today the USP provides 81 standardised graphic images that are widely adopted globally to convey essential medication information, including dosage regimen, usage, precautions, and warnings to both patients and their caregivers (Vaillancourt, Khoury, et al., 2018). Pharmaceutical pictograms enhance patients’ comprehension of medication instructions, supporting accurate adherence and promoting safe use (Paşa et al., 2024).

Evidence from existing studies indicates that pictograms positively influence medication therapy and support the management of chronic conditions such as diabetes mellitus, hypertension, and tuberculosis, ultimately improving treatment outcomes and patients’ quality of life (Chmielewska-Ignatowicz et al., 2021; Merks et al., 2021). Globally, pharmaceutical pictograms have been successfully implemented in various countries, demonstrating significant benefits for patient safety and medication adherence. A study conducted in Belgium within an oncology pharmacy setting showed that pharmacists developed 53 pictogram cards for medication history, adverse effects, drug interactions, and supportive care. After expert review, 52 cards were validated (mean score 7.37 ± 1.2), showing that pictograms can effectively overcome language barriers and improve pharmaceutical care (Clarenne et al., 2025). In Canada, a study assessed 58 participants aged 65 years and older. At baseline, 10 pictograms met the International Standard Organization (ISO) comprehensibility threshold, and after 4 weeks, once meaning had been explained and tested, 13 pictograms were correctly recalled (Vaillancourt et al., 2019). Similarly, countries such as Singapore and Hong Kong have adopted pictograms to cater to multilingual and diverse patient populations, improving understanding of prescriptions, especially among elderly patients and those facing language barriers (Malhotra et al., 2022; Vaillancourt, Zender, et al., 2018).

The implementation of pharmaceutical pictograms in low- and middle-income countries (LMICs) faces significant challenges, including limited regulatory mandates, resistance from pharmaceutical companies due to cost concerns, and limited awareness among healthcare professionals. Without government incentives or legal enforcement, pharmaceutical companies and healthcare providers have little motivation to prioritise pictograms (Heyns et al., 2021). In Pakistan, conveying medication instructions remains a significant challenge due to issues such as patient burden, low patient-to-healthcare provider ratio, diverse linguistic backgrounds, and generally low health literacy within the population (Saqib et al., 2018). These challenges frequently lead to AEs, poor adherence, and threats to patient safety, ultimately intensifying the strain on the healthcare system. Typically, in Pakistan, medication-related instructions are communicated verbally upon dispensing by medical doctors, pharmacists, or pharmacy staff, yet patients often find it hard to remember this information. Additionally, written prescriptions and instructions are not customised to meet patients’ needs, which further hinders comprehension. This absence of clear and effective communication contributes to non-compliance with prescribed treatment plans, resulting in extended hospitalisations, increased healthcare costs, and obstacles in managing health conditions (Saqlain et al., 2019; Shoaib, 2023). Despite the global success of pharmaceutical pictograms in addressing such challenges, their adaptation in Pakistan is minimal, exposing patients to the risk of misinterpreting vital medication instructions. This qualitative study marks the first of its kind in Pakistan, aiming to gather perspectives from health regulators, pharmaceutical manufacturers, and healthcare professionals about the implementation of pharmaceutical pictograms.

## Methodology

### Study setting

This study was conducted across all provinces of Pakistan, including major cities selected based on their population size. Lahore, Bahawalpur, Rawalpindi, and Islamabad were included, with the first three cities representing Punjab and Islamabad serving as the federal capital territory. In Balochistan, Quetta was selected, while Peshawar was chosen from Khyber Pakhtunkhwa (KPK). In Sindh, the focus was on Karachi, Pakistan’s largest city, and Sukkur. The selection of cities was primarily driven by population distribution, with Punjab contributing more cities due to its higher population density, and similar considerations applied to the other provinces (Naveed & Shah, 2024).

### Study design and study participants

This study followed a qualitative exploratory study design, utilising in-depth, face-to-face interviews with experienced participants, each having a working experience of more than two years in their respective fields (Braun & Clarke, 2006). The study was informed by a constructivist paradigm, which assumes that reality and meaning are socially constructed. This perspective was considered suitable as the aim was to explore stakeholders’ subjective interpretations rather than to establish universal truths. The reporting of the study was guided by the Consolidated Criteria for Reporting Qualitative Research (COREQ) to ensure methodological clarity, transparency, and completeness.

The interviews were conducted with individuals representing three main groups: health regulators, healthcare professionals, and pharmaceutical manufacturers. This diverse selection of participants provided valuable insights into the roles of regulation, clinical practice, and pharmaceutical manufacturing, highlighting stakeholders’ perspectives on pharmaceutical pictograms and their impact on healthcare in Pakistan.

### Interview schema development

The interview schema was specifically designed for each stakeholder group to address the study rationale (See supplementary file 1–3). This design process was informed by a comprehensive literature review and consultations with experts (Ferreira-Alfaya, 2024; Paré & Kitsiou, 2017). Before the final round of interviews, pilot interviews were conducted with two representatives from each stakeholder group to ensure the validity, uniformity, and clarity of the study protocols. In line with the constructivist stance, interviews were semi-structured and flexible, allowing participants to construct and share their own meanings. These ranged from general inquiries to more focused questions that directly aligned with the study objectives. This approach ensured that the questions were relevant to the experiences and expertise of each participant, fostering an environment where valuable insights could be gathered to address the key themes of the study.

### Data collection

The data was collected in distinct phases between May and July 2024. Initially, in May 2024, interviews were conducted with Drug Regulatory Authority of Pakistan (DRAP) health regulators and representatives from pharmaceutical companies. Subsequently, in June 2024, health regulators from health departments participated in the study, followed by interviews with hospital and community pharmacists in July 2024. The ‘saturation point’ criterion was employed to ensure the adequacy of the sample size (Saunders et al., 2018). Data saturation was determined when no new themes or insights emerged from the interviews, and the information obtained became repetitive across participants. Two more interviews were conducted to confirm the data saturation (Guest et al., 2020; Naeem et al., 2024).

Participants were gathered through a systematic two-stage process using purposive convenience sampling methods (Ahmed, 2024). In the first stage, selected participants were purposefully contacted during their working hours (8:00 AM to 4:00 PM) at their respective workplaces. Participants who met the eligibility criteria were invited to join the study. In the second stage, those who provided written informed consent were scheduled for interviews at a mutually convenient time and location. Purposive convenience sampling was adopted due to the limited availability and demanding schedules of the study participants. This approach allowed for timely and feasible data collection from willing and accessible participants, while also ensuring the inclusion of information-rich individuals appropriate for the exploratory nature of the study. Interview procedures and data collection details are reported in line with COREQ guidelines to enhance transparency.

Every interview was carried out in Urdu, the national language of Pakistan. The semi-structured interviews were led by the author, with each session audio-recorded and transcribed verbatim to ensure accuracy and reliability.

### Data analysis

Data were analyzed thematically using an inductive thematic analysis, consistent with the constructivist paradigm, with emphasis on participants’ subjective interpretations rather than predetermined categories. The recordings of all interviews were listened to multiple times and transcribed verbatim by the first author. Transcripts were translated into English and checked through a forward – backward translation of approximately 20% of the transcripts to ensure accuracy (Atif et al., 2019). To become fully familiar with the data, transcripts were read repeatedly. Manual coding was carried out by all authors. Words, phrases, and sentences relevant to the study objectives were labelled, and the data were inductively coded to organise them into meaningful segments. Patterns identified from early interviews were used to refine and guide the coding of subsequent transcripts, ensuring consistency and depth in the analysis. Initial open coding was followed by focused coding, where relationships among codes were explored on the basis of similarity, difference, frequency, and relevance. Final inductive codes were grouped into categories, which were then refined into broader themes and subthemes through iterative team discussions. Transcripts, codes, and categories were revisited multiple times before finalising the themes. Regular team meetings ensured consensus, and any disagreements were resolved by the senior author (MA). The hierarchy of findings was determined according to the relative frequency of themes, which were then presented in the results.

To ensure rigour, multiple strategies were applied. Credibility was supported by interviewing diverse stakeholders and presenting verbatim quotations to capture their views accurately. Transferability was addressed through purposive sampling and a detailed description of the study setting. Trustworthiness and dependability were enhanced by following COREQ guidelines, documenting analytic steps, and involving more than one researcher in reviewing the coding process.

### Ethical approval

Ethical approval for the study was obtained from the Pharmacy Research Ethics Committee (Reference: 167-2023-/PREC, dated Feb 09, 2023). Written informed consent was obtained from all study participants after explaining the aims and objectives of the study. Participants’ identities were kept confidential by assigning unique identification codes.

## Results

In total, 57 respondents participated in the study, including 13 doctors, 15 pharmacists, 15 health regulators, and 14 manufacturers. During the interview process, eight target participants (12% refusal rate) refused to participate in the study due to their busy work schedules and/or lack of interest. The study participants comprised males and females, with men representing the majority of the group. The age of participants ranged from 32 to 55 years (Mean = 40.1). The duration of interviews ranged from 24 to 47 min (Mean = 34.3). The demographic characteristics of all the respondents are provided in Table 1.

**Table 1.** Characteristics of study participants.

Stakeholders	Designation	Gender	Age (years)	Interview duration (Min.)
Healthcare professionals				
Doctor 1	Consultant	Male	35	35
Doctor 2	General Physician	Male	39	39
Doctor 3	AMS^1^	Female	41	37
Doctor 4	Pulmonologist	Male	38	30
Doctor 5	SMO^2^	Female	32	28
Doctor 6	Cardiologist	Male	39	32
Doctor 7	SMO^2^	Female	34	35
Doctor 8	Psychiatrist	Male	42	37
Doctor 9	General Physician	Female	45	39
Doctor 10	General Physician	Male	43	40
Doctor 11	Cardiologist	Male	47	34
Doctor 12	General Physician	Male	39	37
Doctor 13	Urologist	Male	44	29
Pharmacist 1	Hospital Pharmacist	Male	35	32
Pharmacist 2	Senior Pharmacist	Male	39	35
Pharmacist 3	Chief Pharmacist	Male	41	38
Pharmacist 4	Senior Pharmacist	Female	38	28
Pharmacist 5	Clinical Pharmacist	Male	32	37
Pharmacist 6	Manager Pharmacy Operations	Male	39	39
Pharmacist 7	In-charge Pharmacist	Female	34	30
Pharmacist 8	Hospital Pharmacist	Male	42	27
Pharmacist 9	Hospital Pharmacist	Male	45	29
Pharmacist 10	Clinical Pharmacist (ID)^3^	Male	41	30
Pharmacist 11	Hospital Pharmacist	Female	39	33
Pharmacist 12	Hospital Pharmacist	Female	37	38
Pharmacist 13	Hospital Pharmacist	Female	39	39
Pharmacist 14	Senior Pharmacist	Male	41	32
Pharmacist 15	Hospital pharmacist	Female	40	24
Health Regulators				
Regulator 1	Drug Inspector	Male	41	39
Regulator 2	Deputy Director, DRAP^4^	Female	44	36
Regulator 3	Director, DRAP^4^	Male	55	45
Regulator 4	Drug Inspector (IDHD)^5^	Male	43	35
Regulator 5	Deputy Director	Female	41	39
Regulator 6	Drug Controller	Male	43	32
Regulator 7	Director CDL^6^, Karachi	Male	55	47
Regulator 8	Chief Drug Inspector	Male	47	40
Regulator 9	Chief Executive Officer	Male	40	38
Regulator 10	Drug Inspector	Female	38	35
Regulator 11	Deputy Director, DRAP^4^	Male	39	33
Regulator 12	Deputy Drug Controller	Male	42	31
Regulator 13	Deputy Director, DRAP^4^	Male	39	30
Regulator 14	Drug Inspector	Male	38	29
Regulator 15	Deputy Drug Controller	Male	37	33
Pharmaceutical Manufacturers				
Manufacturer 1	Product Manager	Male	35	32
Manufacturer 2	HR head	Male	39	36
Manufacturer 3	Operating Officer	Male	32	40
Manufacturer 4	CEO^7^	Male	49	30
Manufacturer 5	General Manager	Male	40	35
Manufacturer 6	HR^8^ head	Male	48	29
Manufacturer 7	General Manager	Male	39	28
Manufacturer 8	Head of Factory Operations	Male	35	35
Manufacturer 9	General Manager	Male	33	38
Manufacturer 10	Factory Head	Male	39	37
Manufacturer 11	HR^8^ Head	Male	36	32
Manufacturer 12	Manufacturing Manager	Male	41	39
Manufacturer 13	Product Manager	Male	40	42
Manufacturer 14	Manufacturing Associate	Male	38	31

^1^ Additional Medical Superintendent, ^2^ Senior Medical Officer, ^3^Ibfectious Disease, ^4^Drug Regulatory Authority of Pakistan, ^5^Islamabad Drug Health Department, ^6^Central Drug Laboratory, ^7^Chief Executive Officer, ^8^Human Resource Head.

**Figure 1.**
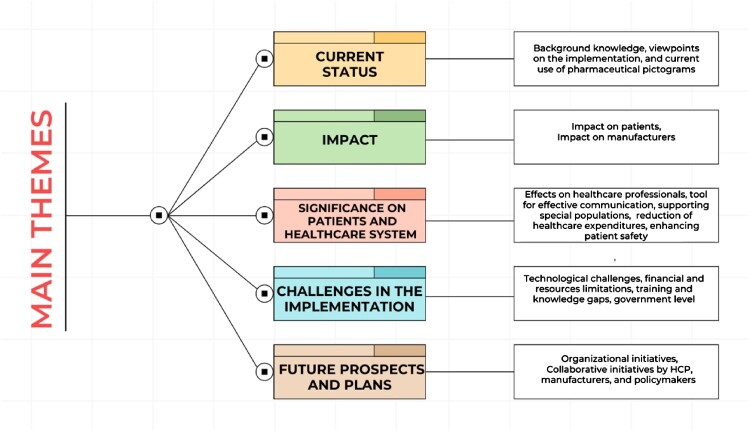
Summary of the themes.

From a saturated pool of information, five key themes were extracted including the current status of pharmaceutical pictograms, the impact of pharmaceutical pictograms, the significance of pharmaceutical pictograms on patients and the healthcare system, challenges in the implementation of pharmaceutical pictograms, and future prospects and plans regarding pharmaceutical pictograms, as shown in Figure 1.

### Theme 1: current status of pharmaceutical pictograms

#### Background knowledge about ‘pharmaceutical pictograms’

**Figure 2.**
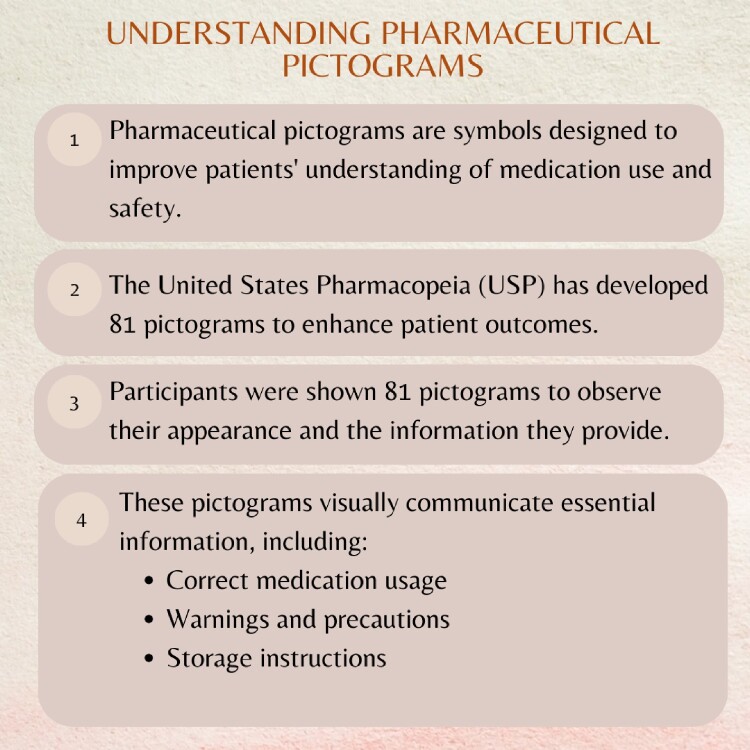
Understanding pharmaceutical pictograms.

Among the participants, the majority of doctors (10 out of 13) and pharmacists (11 out of 15) demonstrated a better understanding of pictograms. However, their knowledge of the standardised system of pharmaceutical pictograms was limited. Likewise, a notable proportion of regulators (10 out of 15) and manufacturers (10 out of 14) were familiar with pictograms but lacked awareness of the established standard framework. For those who were previously unaware, the concept was introduced through explanations and visual demonstrations of pictograms (see Figure 2), enabling them to comprehend the idea and its potential applications more effectively.

#### Viewpoints on the implementation of pharmaceutical pictograms

The majority of the respondents (52 out of 57) expressed a positive attitude towards the implementation of pharmaceutical pictograms within the healthcare system, emphasising their potential to significantly enhance patient understanding and safety. Some respondents further highlighted that limited discussions among colleagues and stakeholders hinder the development of effective strategies for implementing pictograms.

#### Current use of pharmaceutical pictograms

Around half of the doctors (7 out of 13) and pharmacists (8 out of 15) reported small-scale pictogram-based interventions in hospitals to help patients understand their medications better. These efforts aimed to enhance patient comprehension and make medication use easier. Some participants discussed their experiences and observations regarding the current use of pharmaceutical pictograms. The subthemes, categories, and exemplar quotations of Theme 1 are shown in Table 2.

**Table 2.** Theme 1- current status of pharmaceutical pictograms (subthemes, categories, and exemplar quotations).

Theme 1: current status of pharmaceutical pictograms		
Subthemes	Categories & Subcategories	Quotations
Background knowledge about ‘Pharmaceutical Pictograms’	Understanding of the term ‘Pharmaceutical Pictograms’	*‘I don't have much of a specific idea about this. I've learned a little about it in the syllabus, but I've never seen it practically implemented anywhere.’* (Doctor 4) ‘*Yes, I know. Pharmaceutical pictograms essentially involve counseling patients through pictures. It means explaining medications to patients using images and showing them how to take the medicine. Along with symbols, sign language, and pictures, there will also be written instructions. These will indicate whether the medicine should be taken before or after meals or administered via a specific route.’* (Pharmacist 9) ‘*I have heard about the term ‘pictograms,’ but I don’t have extensive knowledge about it.’ (Manufacturer 3)* ‘*Yes, a little bit familiar but not deeply understanding it. I have also been to the United States of America (USA), the United Kingdom (UK), and the United Nations. However, it can’t be used in Pakistan, but I don’t know the reasons. But it's not widely used in third-world countries.’* (Regulator 5).
	Understanding of standard systems of pharmaceutical pictograms	*‘I have not previously come across these standards; this is the first time I am being informed about them*.’ (Doctor 2) ‘*During my time abroad, I learned about the USP and the FIP, but I didn't have in-depth information about its standards*.’ (Regulator 6) ‘*I had not encountered these standards previously; this is new information for me*.’ (Manufacturer 3)
Viewpoints on the implementation of pharmaceutical pictograms	A positive outlook on implementation	*‘I think this should be implemented, not just for the patients but also for healthcare administrators like nurses, staff, and doctors. They should also have pictograms. These should be included with the medications. Whenever medications are dispensed to us, there should be pictograms with them, even if they are small in size.’* (Manufacturer 9) ‘*I firmly believe that launching this initiative will lead to considerable progress within the next two to three years, and people will start to grasp this idea. While there may be some difficulties in getting started and embracing this change at first, it represents a beneficial advancement for the healthcare industry and patient safety.’* (Pharmacist 11)
	Limited discussion with other colleagues	*‘We don’t have such a type of talk. We mainly deal regulatory side. We hadn’t seen such an idea before.’* (Regulator 3) ‘*Without regular discussions, manufacturers may struggle to develop effective strategies for implementing pictograms, leading to inconsistent practices across products.’* (Manufacturer 8)
Current use of pharmaceutical pictograms	Interventions related to pictorial labels	*‘It’s not like a proper intervention, maybe more of a small-scale intervention. Because when we give medicine at discharge, sometimes we draw symbols ourselves for the patients to help them understand. For example, if we need to explain to a patient whether to take the medicine in the morning or at night, we might draw a sun or a moon on it. But at a policy level or regularly, we haven’t used pictograms. At Shifa, we had an intervention with Rotacap because there was an issue where a patient was taking an oral medicine instead of an inhaled one. We had a picture added to the label to address this. However, since I left Shifa, I don’t know the outcome of that intervention.’* (Doctor 11) ‘*Implementing interventions that involve user testing of pictorial labels is crucial for gathering insights on how well they communicate necessary information.’* (Manufacturer 7)

### Theme 2: the impact of pharmaceutical pictograms

All respondents stated that pharmaceutical pictograms will have a significant impact on various sectors of healthcare, affecting patients and pharmaceutical manufacturers.

#### Impact on patients

Many doctors (11 out of 13) stated that pictograms would simplify complex medication instructions, particularly for those facing limited literacy or language barriers. They believed this would improve patients’ understanding, increase treatment adherence, and ultimately optimise patient care.

#### Impact on manufacturers

The majority of the regulators (13 out of 15) and manufacturers (10 out of 14) also pointed out that pharmaceutical manufacturers might face higher production costs and investment in design, but they recognised the benefits, including an improved brand image and greater patient loyalty. Overall, these insights suggested that the widespread use of pharmaceutical pictograms would significantly enhance communication, safety, and patient satisfaction within the healthcare system. The subthemes, categories, and exemplar quotations of Theme 2 are shown in Table 3.

**Table 3.** Theme 2- the impact of pharmaceutical pictograms (subthemes, categories, and exemplar quotations).

Theme 2: the Impact of pharmaceutical pictograms		
Subthemes	Categories & Subcategories	Quotations
Impact on patients	Optimized patient care	*‘Pictograms turn complex medication instructions into simple, clear visuals, empowering patients with limited resources to follow their prescriptions correctly. By using universally recognized symbols, they break down complex concepts such as dosage, timing, method of administration, and potential side effects into simple images, making it easier for patients to comprehend and follow their prescriptions accurately. This visual approach not only enhances medication adherence but also reduces the risk of errors, improves patient safety, and empowers individuals, particularly those with limited access to healthcare resources, to manage their medications confidently and independently.’* (Doctor 6) ‘*When patients understand their medications better through pictograms, they’re more likely to take them correctly and improve overall outcomes.’* (Pharmacist 2)
Impact on manufacturers	Increased production costs and investment in design	*‘For the successful implementation of pictograms in healthcare, it’s crucial for manufacturers and regulatory bodies to collaborate effectively. If a government body mandates pictograms, this could streamline the implementation process. However, the cost of printing on aluminum foil poses a challenge, as it is already expensive, and adding pictograms will increase ink usage. The ink requirements can vary significantly based on design, impacting manufacturing costs. Manufacturers also play a vital role in this initiative by ensuring that pharmacies display charts to guide patients. In hospital settings, nurses can educate patients about medication usage, fostering a chain of knowledge transfer within the community.’* (Regulator 9)
	Enhance brand image and patient trust	*‘Manufacturers that incorporate pictograms on their labels can enhance their reputation for providing patient-centered care, thereby cultivating trust and loyalty among consumers.*’ (Regulator 7) ‘*For manufacturers, integrating pictograms into their packaging is a step toward fostering a more patient-centric image, enhancing their brand’s reputation in the market.’* (Manufacturer 5)

### Theme 3: significance of pharmaceutical pictograms on patients and the healthcare system

Almost all respondents emphasised that pharmaceutical pictograms could play a crucial role in enhancing patient safety and improving the overall healthcare system.

#### Enhance patient safety

Many participants (51 out of 57) highlighted that pictograms would simplify medication instructions, directly reducing the likelihood of medication errors and thereby improving patient safety, especially for those with limited literacy or language skills.

#### Reduction of healthcare expenditures

Most respondents (45 out of 57) indicated that implementing pharmaceutical pictograms could lead to a reduction in healthcare expenditures. They reported that it may decrease preventable errors and hospital readmissions, ultimately resulting in cost savings for both patients and the healthcare system.

#### Supporting special populations

The majority of the doctors (12 out of 13), pharmacists (11 out of 15), and some regulators (7 out of 15) also discussed the potential benefits of pictograms for special populations, such as patients with sensory impairments, including those with visual or hearing disabilities, as well as children and elderly patients. However, the manufacturers did not provide any viewpoints on this matter.

#### Pictograms as a tool for effective communication

Many respondents stated that pictograms can be effectively implemented as a communication tool in healthcare settings. They emphasised that their use could help overcome literacy and language barriers, improving patient understanding and adherence to medication instructions.

#### Effects on healthcare professionals

Around half of the doctors (7 out of 13), pharmacists (8 out of 15), and regulators (7 out of 15) also discussed how pictograms benefit healthcare professionals by improving medication handling, reducing workload, and enhancing patient satisfaction and trust. The subthemes, categories, and exemplar quotations of Theme 3 are shown in Table 4.

**Table 4.** Theme 3- significance of pharmaceutical pictograms for patients and the healthcare system (subthemes, categories, and exemplar quotations).

Theme 3: significance of pharmaceutical pictograms for patients and the healthcare system		
Subthemes	Categories & Subcategories	Quotations
Enhancing patient safety	Improving medication adherence and compliance	*‘Especially in populations like pediatric or elderly patients, using pictograms can be very helpful. For attendants of patients or the elderly themselves, pictograms can significantly aid in ensuring adherence and proper administration of medications. When patients have a visual representation, it makes it easier to understand and follow the instructions correctly.’* (Pharmacist 8)
	Reducing medication errors	*‘Pharmaceutical pictograms provide a visual guide for patients regarding their medication, offering instructions on how, when, and with whom to take their medication. In my view, their significance lies in facilitating patients to ensure they take the right dose at the right time and in the right way. As a hospital or clinical pharmacist, where direct patient contact is common, you can guide patients on proper medication use. Even community pharmacists can play a role in this. Through these efforts, medication errors can be reduced, and adverse drug reactions (ADRs) minimized.’* (Regulator 5)
	Improved health outcomes	*‘By improving comprehension, pictograms can directly enhance medication adherence. When patients clearly understand when and how to take their medications, they are more likely to follow the treatment plan accurately. This, in turn, reduces the chances of errors, increases safety, and ultimately improves health outcomes. So yes, using pictorial diagrams can significantly boost adherence.’* (Doctor 12) ‘*The introduction of pictograms in patient instructions has been shown to lower complications from incorrect medication use, leading to enhanced patient safety and better health results.’* (Manufacturer 11)
	Patient comprehension and recall rate	*‘The benefit of a pictogram is that if it has a picture, the patient can easily recall when to take the medicine, without needing it written down. Usually, we write instructions for patients, like taking the medicine in the morning, afternoon, or evening, or before and after meals. However, if a label or sticker is attached to the medicine, it greatly aids the patient's memory and ensures they take it correctly.’* (Doctor 7)
	Helpful in disease management	*‘Incorporating pictograms into disease management for conditions such as asthma or gastrointestinal issues like Irritable Bowel Syndrome (IBS) and Crohn's disease can significantly improve outcomes. Many patients find it challenging to understand complicated instructions, whether it involves the proper use of inhalers or adhering to dietary guidelines. Pictograms can present the necessary steps straightforwardly and clearly, helping patients to better follow instructions, reduce errors, and manage their health more efficiently.’* (Doctor 10)
Reduction of healthcare expenditures	Decreased hospital readmissions	*‘Pictograms can play a crucial role in reducing hospital readmission rates by enhancing patients’ understanding and adherence to treatment instructions. These visual aids simplify medication guidelines, minimize errors, and significantly lower complications arising from non-compliance. When integrated into healthcare facilities, pictograms can greatly improve patient outcomes and overall treatment effectiveness.’* (Doctor 3)
	Cost savings for patients and the healthcare system	*‘If patients truly understand their medications and treatments, they are less likely to face complications that could lead to extra treatments, emergency visits, or even hospital readmissions. In a pharmacy setting, using tools like pictograms can make a huge difference, helping patients grasp important details without confusion. Ultimately, this not only improves health outcomes but also supports a more cost-effective healthcare system.’* (Pharmacist 7) ‘*Pictograms allow patients to manage their health effectively at home, reducing the need for repeat consultations and saving both patient and system costs. Pictograms support self-management, decreasing the frequency of doctor visits and related costs.’* (Doctor 9)
Supporting special populations	Sensory-impaired patients	*‘Pictograms are very important for special patients. Their effectiveness depends on the type of impairment. Pictograms might not work for vision impairment, but they can be very helpful for the elderly, children, and the deaf.’* (Doctor 13) ‘*For deaf patients, no matter how much verbal counseling we provide about how to use their medications, it becomes useless. Pictograms would be very effective for these patients, making them the gold standard for hearing-impaired individuals. Some blind patients can hear but cannot see their medications; for them, pictograms designed in Braille would be very effective. I believe that introducing pharmaceutical pictograms into the system would be a revolutionary step, ensuring the safety of marginalized populations like those with special needs.’* (Pharmacist 7)
	Pediatrics and geriatrics populations	*‘As a doctor, I have often observed elderly patients struggling to understand their medication instructions. Many of them face memory issues or find it difficult to grasp complex verbal explanations. The same challenge exists for children, especially when their caregivers are not around. Pictograms can serve as a bridge, helping both the elderly and children easily recall instructions. An elderly patient living alone can confidently take their medication correctly just by looking at the pictogram, without waiting for assistance. Similarly, a child can understand a simple visual cue much more easily than a lengthy verbal explanation. This is a simple yet powerful tool that can be highly beneficial for these vulnerable groups.’* (Doctor 5)
Pictograms as a tool for effective communication	Overcoming literacy and language barriers	*‘It could be much easier to explain to the patient. For example, when we inform patients about their medication, especially for admitted patients, it often becomes difficult to explain to discharged patients how to take their medication because of literacy issues, as the literacy rate isn't very high. It’s challenging to explain how and when to take the medicine. I think using pictograms would make it much easier to explain. It would be easier for us and would save time.’* (Doctor 12) ‘*I believe pictograms are a great way to minimize health literacy issues. Language barriers and communication gaps are significant challenges, particularly with patients who speak Sindhi or Balochi, whose native languages differ from ours. We often cannot effectively counsel or educate them, leading to numerous medication errors. If pictograms were available for common medications, it would greatly help patients understand how to use them. By showing them how to use the medication, they are more likely to remember it, which could reduce many health issues.’* (Regulator 8)
Effects on healthcare professionals	Improve medication handling by healthcare professionals	*‘For doctors, by incorporating pictograms into medication handling processes, doctors can quickly and accurately interpret complex drug regimens, reducing the risk of errors in prescribing and administration.’ (Doctor 7)* ‘*Incorporating pictograms into the medical workflow enables doctors to easily comprehend medication regimens, reducing cognitive overload and supporting more accurate handling of medications. As a result, the chances of medication errors such as incorrect dosages or improper administration are significantly minimized, leading to better patient safety and improved treatment outcomes.’* (Pharmacist 9)
	Decrease workload on healthcare professionals	*‘Visual aids like pictograms provide clear, easy-to-understand instructions for patients, decreasing the burden on healthcare providers to explain complex medication regimens multiple times.’* (Regulator 9)
	Improved patient satisfaction and trust in healthcare providers	*‘When patients feel confident they understand their treatment, they are more likely to have a positive experience and adhere to their care plans. This trust also strengthens the patient-provider relationship.’* (Doctor 13)

### Theme 4: challenges in the implementation of pharmaceutical pictograms

Several key challenges were identified by respondents that could pose significant barriers to the successful implementation of pharmaceutical pictograms.

#### Government level

At the government level, a major concern highlighted by many respondents was the lack of recognition and support from authorities. Respondents emphasised that without official endorsement or regulatory mandates, implementing pharmaceutical pictograms would face significant challenges. Specifically, 14 out of 15 regulators and 11 out of 14 manufacturers reported that the absence of formal regulations makes it difficult to ensure consistent use of pictograms across healthcare settings.

#### Training and knowledge gaps

The majority of the respondents (53 out of 57) highlighted a significant gap in training and knowledge among healthcare professionals, emphasising the need for proper education and awareness to ensure the correct and effective use of pictograms in healthcare settings.

#### Financial and resource limitations

Many respondents also cited financial and resource constraints as significant obstacles to implementing pharmaceutical pictograms. They highlighted that the creation, printing, and distribution of pictograms would require substantial investment, which could be particularly challenging in resource-limited settings.

#### Technological challenges

Another challenge mentioned was the integration of pictograms into existing medication labelling systems. Many health regulators (12 out of 15) indicated that modifying established packaging and labelling processes could be complex, necessitating significant changes and retraining of staff. While these challenges were acknowledged, participants also recognised that with the right strategies, support, and investment, the full benefits of pharmaceutical pictograms could be realised. The subthemes, categories, and exemplar quotations of Theme 4 are shown in Table 5.

**Table 5.** Theme 4- challenges in the implementation of pharmaceutical pictograms (subthemes, categories, and exemplar quotations).

Theme 4: challenges in the implementation of pharmaceutical pictograms		
Subthemes	Categories & Subcategories	Quotations
Government level	No support from the government	*‘If the government doesn't endorse pictograms, pharmaceutical companies are hesitant to invest in them. Clear policy support could drive meaningful change across the industry, but we’re currently lacking that guidance. This is a significant challenge for implementing pictograms in Pakistan.’* (Doctor 6) ‘*Government support is critical for widespread adoption. Without it, only a handful of hospitals and clinics can implement pictograms effectively, leaving most patients without this essential visual aid. The government should take proper steps to implement them in healthcare settings to improve patient outcomes.’* (Manufacturer 4)
	Absence of a regulatory framework	*‘In Pakistan, there is currently no practical implementation of using pictograms for patients. There are no rules or practices in place for this, and DRAP and the regulations in Pakistan do not include any such provisions.’* (Regulator 14) ‘*Without a regulatory framework, their adoption in healthcare systems is often limited. There are no established guidelines mandating us to incorporate pictograms in patient information leaflets. Policies and standards must be in place to maximize the potential of visual aids in enhancing patient safety.’* (Manufacturer 5)
Training and knowledge gaps	Lack of healthcare professional training on pharmaceutical pictograms	*‘We have to conduct some training programs for doctors and try to implement them at the hospital level, from this compliance will increase to some extent. This is also a barrier to implementing pharmaceutical pictograms in the system.’* (Doctor 8) ‘*Without adequate training on the use of pharmaceutical pictograms, healthcare professionals may struggle to communicate effectively with patients, particularly those with low literacy levels. Training is essential for maximizing the potential of pictograms as tools for patient understanding and adherence.’* (Regulator 9)
	Lack of awareness among healthcare professionals, drug regulators, and manufacturers	*‘Awareness about pictograms is often low among healthcare professionals and regulators. This lack of understanding makes it harder to support patients with clear, easy-to-follow instructions, especially for those with limited literacy.’ (Doctor 6)* ‘*Drug manufacturers and regulators often fail to recognize the importance of pictograms due to a lack of awareness. However, educating these groups about the benefits of pictograms can greatly improve medication instructions. This is particularly crucial for patients with low literacy or those who struggle with reading, as pictograms offer clear, visual representations that enhance understanding and adherence to medication instructions, ultimately improving patient safety.’* (Regulator 11)
	Lack of collaborative efforts between stakeholders	*‘No matter how much effort we put in, implementing pictograms will remain challenging unless all relevant stakeholders recognize their importance. Progress will be limited unless regulators, doctors, and pharmaceutical companies actively participate in this discussion. While we can take the initiative to promote this effort, it will not be truly effective without concrete actions at every level. Successful implementation requires collaboration across all sectors, but achieving this alignment will be a significant challenge.*’ (Regulator 9)
Financial and resource limitations	Budget constraints	*‘I believe that pictograms can be an effective tool in improving patients’ understanding and reducing hospital readmission rates. However, due to budget issues in our healthcare system, their implementation remains a challenge. The lack of financial resources creates a barrier to prioritizing such interventions.’* (Doctor 6) ‘*Budget constraints pose a major challenge to implementing new patient communication tools, such as pictograms, particularly in settings with limited resources. The cost of production and distribution can be prohibitive, making it difficult for healthcare systems to adopt these tools despite their potential to improve patient understanding and safety.’* (Regulator 5)
Technological challenges	Integration with an existing medication labeling system	‘*The integration of pictograms into existing medication labels faces resistance from stakeholders who are accustomed to traditional labeling practices and may be reluctant to adopt new systems.’* (Doctor 6) ‘*Integrating pictograms with current labeling systems may face compatibility challenges, requiring careful planning to align with existing technologies and workflows. Addressing these issues involves adapting design, software, and printing processes to support pictogram formats while maintaining regulatory compliance and clarity for end-users.’* (Pharmacist 9)

### Theme 5: future prospects and plans regarding pharmaceutical pictograms

Looking ahead, all participants expressed positive views on the prospects of pharmaceutical pictograms, highlighting several key areas where growth is anticipated.

#### Organisational initiatives

The majority of the respondents highlighted that the successful implementation of pharmaceutical pictograms would require careful planning of future budgets and timelines. They emphasised the need for developing standardised guidelines and protocols to ensure consistent use across healthcare settings. Education and awareness campaigns were seen as crucial for both healthcare professionals and patients. Specifically, 11 out of 13 doctors and 13 out of 15 health regulators stressed that training programmes are essential to maximise the effectiveness of pictograms, enhance patient understanding, and improve adherence to medication instructions. Respondents also noted that progressive adoption, supported by organisational initiatives, could help integrate pictograms into routine practice in a structured and sustainable manner.

#### Collaborative initiatives by healthcare professionals, manufacturers, and policymakers

Collaborative efforts were widely supported, with many participants calling for a unified approach from healthcare professionals, pharmaceutical manufacturers, and policymakers to shape the future integration of pictograms. They believed that such collaboration could accelerate the process, address existing challenges, and ensure that pictograms become a standardised part of healthcare practices, ultimately improving patient safety, comprehension, and outcomes. The subthemes, categories, and exemplar quotations of Theme 5 are shown in Table 6.

**Table 6.** Theme 5- future prospects and plans regarding pharmaceutical pictograms (subthemes, categories, and exemplar quotations).

Theme 5: Future prospects and plans regarding pharmaceutical pictograms		
Subthemes	Categories & Subcategories	Quotations
Organisational initiatives	Future budget and timelines for the implementation of pharmaceutical pictograms	*‘Effective implementation of pharmaceutical pictograms requires a well-defined budget that accounts for design, production, and training costs to ensure sustainable adoption in the long term.’* (Doctor 7) ‘*Regular consultations with stakeholders throughout the budgeting process can help identify potential financial barriers early, allowing for proactive solutions to keep timelines on track.’* (Manufacturer 4)
	Developing guidelines and protocols	*‘Drafting clear guidelines for pictogram implementation is essential to ensure that they are used consistently across all healthcare settings, improving understanding for all patients.’* (Regulator 8) ‘*All manufacturing companies in Pakistan are required to follow DRAP's instructions and regulations. If we don't comply, we face problems from DRAP. We would be in favor of adding these to boxes or leaf inserts for the benefit of patients, but without clear instructions from DRAP, we cannot move forward. As you can see, this box was designed according to DRAP's directions. It’s essential to wait for DRAP’s approval and guidelines to ensure that we remain compliant while also making the process more patient-friendly.’* (Manufacturer 9)
	Education and awareness campaigns	*‘Education is key to the successful implementation of pictograms; healthcare providers and patients must understand their purpose and how to use them effectively.’* (Doctor 13) ‘*Awareness programs are essential as they provide a basic understanding. Pharmacists working in hospital pharmacies should be knowledgeable about medication timings, such as after meals, before meals, or 30 min prior. Regular weekly meetings at the hospital level could facilitate the sharing of this knowledge among pharmacists. This way, they can educate technicians and nurses, ultimately reducing medication errors.’* (Regulator 7)
	Progressive Adoption of Pictogram	*‘Small-scale interventions, such as introducing pictograms gradually, can lead to immediate improvements in patient understanding and compliance, showcasing the benefits of pictograms without requiring large investments.’* (Doctor 9) ‘*By starting with small projects and making changes one step at a time, both staff and patients can gradually become comfortable with using pictograms. This approach allows everyone to adjust to the new system without feeling overwhelmed. As they see the benefits of pictograms in helping them understand medication instructions better, they are more likely to embrace these changes in their daily routines. It’s a way to make sure that everyone is on board and that the transition to using pictograms is smooth and effective.’* (Manufacturer 5)
Collaborative initiatives by healthcare professionals, manufacturers, and policymakers	Plan to promote pictograms and discuss them with colleagues	*‘I will download the USP pictograms, share them with my colleagues, and arrange educational programs for their implementation if there's an opportunity. I am committed to doing whatever I can to promote this.’* (Pharmacist 11) ‘*I will try to introduce the concept of pharmaceutical pictograms in any upcoming official meetings at the hospital. My goal is to demonstrate how these pictograms can play a significant role in improving patient safety and adherence. This will help raise awareness among higher hospital authorities and encourage them to consider possible steps to enhance patient safety.’* (Regulator 9)

## Discussion

Pharmaceutical pictograms have emerged as a valuable tool for improving patient comprehension and promoting proper medication use, particularly in populations with low literacy levels or linguistic barriers. This study aimed to explore the perspectives of multiple key stakeholders on implementing the pharmaceutical pictograms in Pakistani healthcare settings. The participants expressed strong support for the implementation of pharmaceutical pictograms, recognising their potential to enhance patient outcomes. They emphasised the necessity of standardised pictograms accompanied by clear policy guidelines to facilitate effective patient communication.

Most of the respondents were familiar with the general concept of pictograms; their understanding of standardised systems, such as those developed by USP and FIP, was limited. This knowledge deficit is likely due to the lack of integration of pictograms into medical and pharmacy curricula in Pakistan, leaving healthcare professionals unfamiliar with their practical applications and significance (Memon et al., 2024). In contrast, evidence from developed countries indicates that healthcare professionals typically possess comprehensive knowledge of pharmaceutical pictograms, supported by established guidelines and active regulatory bodies (Ferreira-Alfaya et al., 2024).

Despite these gaps, the majority of the participants strongly supported the implementation of pharmaceutical pictograms, emphasising their potential to improve patient understanding, particularly in populations with low literacy or language barriers. These views are consistent with international studies demonstrating that pictograms simplify complex medical instructions, promote patient safety and medication adherence, and reduce the risk of errors in drug administration (Ferreira-Alfaya, 2024; Pires, 2024). The study participants also highlighted that its implementation was reported to be particularly beneficial for vulnerable populations, including pediatric, geriatric, and sensory-impaired patients, as it enhances comprehension and recall, consistent with findings from previous studies (Lin et al., 2021). The majority of the participants highlighted that although increased production costs and design investment in pictograms could impact manufacturers, it could enhance their brand image and patient trust. These conclusions are further supported by the findings of Jami et al, whose research similarly demonstrated that pharmaceutical pictograms contribute to both patient safety and manufacturer credibility, reinforcing their importance in the healthcare system (Jami et al., 2025).

The study findings showed that the implementation of pharmaceutical pictograms in Pakistan’s healthcare system can significantly improve medication adherence, particularly for chronic diseases such as tuberculosis (TB), hypertension, and hepatitis, which require long-term therapy. In Pakistan, low literacy rates, language barriers, and lack of patient awareness often lead to misunderstandings of medication instructions, contributing to poor adherence and treatment failure. Given the fact that Pakistan has a fragmented healthcare system where uptake of modern technologies is minimal, pharmacists are not available in most of the healthcare settings, and the physician-to-patient ratio is low, the implementation of pharmaceutical pictograms can significantly improve the patient’s understanding of disease, thereby improving target outcomes (Shoaib, 2023). The use of pictograms in such an overburdened healthcare system is very relevant to conditions such as tuberculosis, hypertension, stroke, asthma, diabetes, etc (Saqlain et al., 2019). Additionally, in a culturally diverse population where multiple languages are spoken, pictograms can serve as a universal communication tool, reducing dependence on written instructions and healthcare professionals for repeated guidance (Guerrero, 2022).

However, the implementation of pharmaceutical pictograms in Pakistan faces several systemic challenges, as highlighted by study participants. Regulatory gaps were identified as a major barrier, including limited government recognition, insufficient training of healthcare professionals, and a lack of collaborative efforts among key stakeholders. These observations align with findings from international studies conducted in Iran, South Africa, and Portugal, which also reported that inadequate government endorsement and insufficient provider education hindered the adoption of pictograms (Dowse, 2021; Faustino et al., 2021). The study participants stated that financial and resource limitations were significant barriers in Pakistan, as implementing pharmaceutical pictograms requires investments in training programmes and redesigning packaging. These financial limitations not only slow down the adoption process but also create an imbalance in the accessibility and implementation of pictograms across various healthcare settings, particularly in countries with low literacy levels (Dowse, 2022).

To address the barriers identified in this study, several strategies were proposed to facilitate the effective implementation of pharmaceutical pictograms in Pakistan. Participants emphasised the need for proactive initiatives by relevant authorities, including allocating future budgets and establishing timelines for pictogram adoption. Developing clear guidelines and standardised protocols was also highlighted as essential to ensure consistent and uniform use across the healthcare system, consistent with findings from studies conducted in Lebanon (Mourad et al., 2023). Additionally, the participants emphasised that education and awareness campaigns could be critical to educating healthcare professionals and patients about the significance and proper use of pictograms. They further stated that collaborative initiatives between healthcare professionals, manufacturers, and policymakers are necessary to create a unified approach and foster the widespread use of pictograms. Pharmacists, in particular, were identified as key facilitators, as their involvement in educating patients, developing, and distributing pictograms could enhance patient comprehension and reduce medication errors, especially among populations with limited literacy or language barriers. These findings align with studies conducted in other countries, such as the Philippines and Nigeria, where similar strategies were suggested to overcome barriers (Abdu-Aguye et al., 2023; Gutierrez et al., 2022). Thus, these proposed solutions align with our study’s findings and reflect an international consensus on promoting pictograms for improving patient safety and healthcare outcomes.

### Impact of findings on policy and practice

The findings of this study highlight the importance of pharmaceutical pictograms in improving medication comprehension and adherence. These insights underscore the need for policymakers to develop standardised guidelines for the design and implementation of pictograms within Pakistani healthcare systems. Integrating pictograms into national health policies can enhance communication between healthcare providers and patients, ultimately improving patient safety and reducing medication-related errors. Furthermore, the study emphasised the significance of training healthcare professionals for the effective use of pictograms, fostering a more patient-centered approach in clinical practice. By addressing these aspects, the findings pave the way for improved health outcomes and a more inclusive process in medication management. Future research should explore the practical feasibility, long-term sustainability, and cost-effectiveness of incorporating pharmaceutical pictograms into healthcare systems.

### Strengths and limitations

This study significantly contributes to the limited body of literature on the concept of implementing pharmaceutical pictograms in Pakistan. A notable strength of this research is that it represents the first of its kind in Pakistan, specifically focusing on the implementation of pharmaceutical pictograms. Another key strength lies in its comprehensive approach, as it includes interviews with various stakeholders, such as doctors, pharmacists, manufacturers, and regulators from major cities across Pakistan. This holistic perspective enabled the collection of diverse viewpoints and provided an in-depth understanding of the potential application of pharmaceutical pictograms within the local healthcare system.

However, our study has some limitations. The convenience sampling method may have constrained the diversity of opinions, possibly overemphasising certain viewpoints. It was used for its practicality and efficiency in accessing relevant stakeholders with direct experience in pharmaceutical pictograms. While it may limit diversity, it provides valuable insights that are useful for participants (Atif et al., 2020). The exploratory qualitative design makes the findings context-specific and not broadly generalisable. However, rigour was enhanced by following COREQ standards. Furthermore, the study did not assess the practical integration, effectiveness, or cost-effectiveness of pharmaceutical pictograms within its scope. Additionally, social desirability bias may have influenced some responses, and the absence of patient perspectives limits the ability to fully capture how pictograms are understood and applied in practice.

## Conclusion

This qualitative study examined the perspectives of key stakeholders, who strongly favoured the implementation of pharmaceutical pictograms, recognising their potential to enhance patient comprehension, adherence, and overall safety in Pakistan. However, their adoption is limited due to insufficient awareness, less policy support, technological issues, and inadequate resources. Addressing these challenges through national policies, professional training, and awareness initiatives is essential for the successful adoption of pictograms in the healthcare system. The study underscores the need for a structured framework to standardise and integrate pharmaceutical pictograms into Pakistan’s healthcare system.

## Supplementary Material

Supplementary_File__1_Clean.docx

No Funding Letter _1.pdf

## Data Availability

The data generated and analyzed during this study are not publicly available due to confidentiality and ethical restrictions. Additional participant quotations supporting the results are provided in the supplementary file 1. Further information may be available from the corresponding author upon reasonable request.
