# Supplementary material for: A qualitative study to explore the perspectives of key stakeholders regarding pharmaceutical pictograms in Pakistan
Source: J Pharm Policy Pract. 2026 Jan 12;19(1):2598481. doi: 10.1080/20523211.2025.2598481 (PMC12798668; doi:10.1080/20523211.2025.2598481)
Supplement: Supplementary_File__1_Clean.docx [file JPPP_A_2598481_SM8686.docx]

**Supplementary File 1**

Additional participant quotations supporting the results have been provided in the supplementary file 1.

**Table 2: Theme 1- Current Status of Pharmaceutical Pictograms (Subthemes, Categories, and Exemplar Quotations)**

| **Theme 1: Current Status of Pharmaceutical Pictograms** | | | |
| --- | --- | --- | --- |
| **Subthemes Categories & Subcategories Quotations** | | | |
| Viewpoints on the implementation of pharmaceutical pictograms | A positive outlook on implementation | *“I have some dentist colleagues who use charts and visual aids to teach patients how to brush properly, employing a proper approach. I believe pictograms are very important for patients and should be implemented to overcome literacy issues, especially in Pakistan where literacy rates are low. I don't see any disadvantages to using pictograms, as long as they are designed according to our culture and language, with clear messages for patients”.* (Doctor 7)  *“From the perspective of a drug regulator, while our mandate is focused on regulating the sale of drugs, I believe there is room for improvement in our packaging and labeling rules. Particularly for over-the-counter (OTC) drugs that are sold without a prescription, incorporating pictograms could be beneficial. If we start by targeting a specific group of medicines, such as OTC drugs, and apply pictograms to them, it could serve as a pilot study. This would allow us to evaluate the effectiveness of pictograms and understand how they could be beneficial in the future”*. (Regulator 5) |  |
|  | Limited discussion with other colleagues | *“I haven't talked about pharmaceutical pictograms with other doctors or pharmacists, nor have I brought it up in any of our official meetings”.* (Doctor 7) |  |
| Current use of pharmaceutical pictograms | Interventions related to pictorial labels | *“During the COVID-19 pandemic, we told patients to use sanitizers, but many didn’t understand. We explained the steps for using hand sanitizer through pictures, and it had a great* out*come; people understood how to use it properly. In the pulmonology OPD, I had a patient whom I advised to quit smoking to prevent lung damage. However, he didn’t grasp how his lungs could deteriorate over the coming years. I showed him pictures depicting what lungs could look like after 10 or 20 years, which were very effective visual aids. I believe pictograms are important not only for medications but also for disease management”.* (Pharmacist 11)  *“We are already using some auxiliary labels and pictograms, like ‘shake well before use,’ for high-alert and life-saving medications. With further study, we can potentially implement more pictograms and provide guidelines for their use. This is an important area in ambulatory pharmacy care, and we plan to take steps toward implementation in the future”.* (Pharmacist 10)  *“We counsel patients in case of use of insulin we have a policy of our institute in pediatrics we gave them leaflets which have pictures to describe about storage of insulin. In this way, we are using it on a small scale”.* (Doctor 12) |  |

**Table 3: Theme 2- The Impact of Pharmaceutical Pictograms (Subthemes, Categories, and Exemplar Quotations)**

| **Theme 2: The Impact of Pharmaceutical Pictograms** | | | |
| --- | --- | --- | --- |
| **Subthemes Categories & Subcategories Quotations** | | | |
| Impact on manufacturers | Increased production costs and investment in design | *“The initial expenses associated with the development and implementation of pictograms may be high, but manufacturers are likely to find these costs manageable if these efforts result in favorable outcomes for patients. Furthermore, manufacturers stand to gain from the long-term benefits that this strategy presents”.* (Manufacturer 5) |  |

**Table 4: Theme 3- Significance of Pharmaceutical Pictograms for Patients and Healthcare System (Subthemes, Categories, and Exemplar Quotations)**

| **Theme 3: Significance of Pharmaceutical Pictograms for Patients and Healthcare System** | | | |
| --- | --- | --- | --- |
| **Subthemes Categories & Subcategories Quotations** | | | |
| Enhancing patient safety | Improving medication adherence and compliance | *“By providing a visual aid, pictograms help patients remember dosing schedules and instructions, improving compliance and reducing instances of missed or incorrect doses. Points out that pictograms can act as memory aids, making it more likely that patients will remember how and when to take their medications”*. (Regulator 2) | |
|  | Patient comprehension and recall rate | *“It is important for patient comprehension, elaboration, and recalling. When they forget after going home, they can look at the symbol and recall how they were counseled about the medicine. That would be an easier and more convenient way for recalling and memorizing in this busy lifestyle”.* (Manufacturer 9) | |
|  | Pediatrics and geriatrics popultions | *“In populations like pediatrics or elderly individuals, pictograms can be very helpful. For children, pictograms can aid in understanding medication instructions, while for elderly patients, pictograms can assist their attendants in ensuring proper medication administration. A visual representation can enhance medication adherence and help ensure the proper medicine administration.”* (Pharmacist 7) | |
| Pictograms as a tool for effective communication | Overcoming literacy and language barriers | *“We have to analyze many things like benefits or policy. In our hospital, most of the patients are poor with minimum literacy rates which will be very beneficial for them. We face difficulty in counseling patients because patients come from other countries like Iran and Afghanistan, pharmaceutical pictograms will helpful in understanding patients in different languages. It will decrease the language barrier, also increase patient compliance and understanding of the patient. So it is a tool for effective communication”.* (Doctor 11) | |
| Effects on healthcare professionals | Decrease workload on HCP | *“The use of pictograms as a patient education tool aids comprehension. Pictograms promote greater patient independence, as individuals can refer to these visuals to reinforce instructions at home. In the long term, this efficiency not only lightens the workload on healthcare providers but also optimizes resources within the healthcare system, ultimately supporting better patient outcomes and adherence”.* (Doctor 7) |  |
|  | Improved patient satisfaction and trust in healthcare providers | *“Pictograms help HCPs communicate more effectively, leading to increased patient satisfaction and trust in their healthcare providers”.* (Pharmacist 9) |  |

**Table 5: Theme 4- Challenges in the implementation of Pharmaceutical Pictograms (Subthemes, Categories, and Exemplar Quotations)**

| **Theme 4: Challenges in the implementation of Pharmaceutical Pictograms** | | | |
| --- | --- | --- | --- |
| **Subthemes Categories & Subcategories Quotations** | | | |
| Government level | Absence of regulatory framework | *“In the absence of a regulatory framework, the effectiveness of patient-centered communication tools, such as pictograms, remains limited. Clear regulations ensure that health information is accessible and comprehensible for all patients”.* (Pharmacist 9)  *“Without clear and standardized guidelines, the effectiveness of pictograms in improving patient safety and medication adherence may be compromised. Consistency in pictogram design and usage across healthcare facilities is essential. Regulatory bodies must establish protocols for the creation, testing, and approval of pharmaceutical pictograms to maintain quality and accuracy in health communication”.* (Doctor 10) | |
| Training and knowledge gaps | Lack of healthcare professional training on pharmaceutical pictograms | *“Pictograms can make medical instructions clearer, but without training, healthcare workers don’t always use them well. Training on pictograms should be a priority to help patients understand how to use their medicines safely”.* (Pharmacist 12)  *“Training would ensure HCPs understand how to use pictograms, making their counseling both consistent and effective. This change may require workshops and ongoing support to help HCPs integrate pictograms into their standard practices”.* (Doctor 10) |  |
|  | Lack of collaborative efforts between stakeholders | *“Effective implementation of pictograms requires collaboration at all levels. Without it, the fragmented approach to patient education hinders clear medication guidance. Without collaboration, implementing pictograms will be very difficult”.* (Doctor 6) |  |

**Table 6: Theme 5- Future Prospects and Plans regarding Pharmaceutical Pictograms (Subthemes, Categories, and Exemplar Quotations)**

| **Theme 5: Future prospects and plans regarding Pharmaceutical Pictograms** | | | |
| --- | --- | --- | --- |
| **Subthemes Categories & Subcategories Quotations** | | | |
| Organizational initiatives | Future budget and timelines for the implementation of pharmaceutical pictograms | *‘Effective implementation of pharmaceutical pictograms requires a well-defined budget that accounts for design, production, and training costs to ensure sustainable adoption in the long term.’* (Doctor 7)  *‘Regular consultations with stakeholders throughout the budgeting process can help identify potential financial barriers early, allowing for proactive solutions to keep timelines on track.’* (Manufacturer 4) | |
|  | Developing guidelines and protocols | *‘Drafting clear guidelines for pictogram implementation is essential to ensure that they are used consistently across all healthcare settings, improving understanding for all patients.’* (Regulator 8)  *‘All manufacturing companies in Pakistan are required to follow DRAP's instructions and regulations. If we don't comply, we face problems from DRAP. We would be in favor of adding these to boxes or leaf inserts for the benefit of patients, but without clear instructions from DRAP, we cannot move forward. As you can see, this box was designed according to DRAP's directions. It’s essential to wait for DRAP’s approval and guidelines to ensure that we remain compliant while also making the process more patient-friendly.’* (Manufacturer 9) | |
|  | Education and awareness campaigns | *‘Education is key to the successful implementation of pictograms; healthcare providers and patients must understand their purpose and how to use them effectively.’* (Doctor 13)  *‘Awareness programs are essential as they provide a basic understanding. Pharmacists working in hospital pharmacies should be knowledgeable about medication timings, such as after meals, before meals, or 30 minutes prior. Regular weekly meetings at the hospital level could facilitate the sharing of this knowledge among pharmacists. This way, they can educate technicians and nurses, ultimately reducing medication errors.’* (Regulator 7) | |
|  | Progressive Adoption of Pictogram | *‘Small-scale interventions, such as introducing pictograms gradually, can lead to immediate improvements in patient understanding and compliance, showcasing the benefits of pictograms without requiring large investments.’* (Doctor 9)  *‘By starting with small projects and making changes one step at a time, both staff and patients can gradually become comfortable with using pictograms. This approach allows everyone to adjust to the new system without feeling overwhelmed. As they see the benefits of pictograms in helping them understand medication instructions better, they are more likely to embrace these changes in their daily routines. It’s a way to make sure that everyone is on board and that the transition to using pictograms is smooth and effective.’* (Manufacturer 5) | |
| Collaborative initiatives by healthcare professionals, manufacturers, and policymakers | Plan to promote pictograms and discuss them with colleagues | *‘I will download the USP pictograms, share them with my colleagues, and arrange educational programs for their implementation if there's an opportunity. I am committed to doing whatever I can to promote this.’* (Pharmacist 11)  *‘I will try to introduce the concept of pharmaceutical pictograms in any upcoming official meetings at the hospital. My goal is to demonstrate how these pictograms can play a significant role in improving patient safety and adherence. This will help raise awareness among higher hospital authorities and encourage them to consider possible steps to enhance patient safety.’* (Regulator 9) |  |
