# Supplementary material for: A qualitative study to explore the perspectives of key stakeholders regarding pharmaceutical pictograms in Pakistan
Source: J Pharm Policy Pract. 2026 Jan 12;19(1):2598481. doi: 10.1080/20523211.2025.2598481 (PMC12798668; doi:10.1080/20523211.2025.2598481)
Supplement: No Funding Letter _1.pdf [file JPPP_A_2598481_SM8685.pdf]

ORIC
The Islamia University of Bahawalpur
Office of Research Innovation & Commercialization (ORIC)
Email, ORIC@IUB.EDU.PK Ph: +92- 9255460

No. 158 / ORIC
Date. 15-05-2025

To Whom It May Concern

It is hereby declared that the manuscript entitled **"A Qualitative Study to Explore the Perspectives of Key Stakeholders Regarding Pharmaceutical Pictograms in Pakistan"** is not funded by any source including national and/or international entities.

This office further regrets to the corresponding author, i.e., **Kanza Arshad**, PhD Candidate, Department of Pharmacy Practice, The Islamia University of Bahawalpur, that the University, in its current capacity, is unable to provide any financial support for the successful publication of the said manuscript.

Kind regards

Additional Director
Office of Research, Innovation and
Commercialization
